# Ramsay Hunt Syndrome in Asymptomatic COVID-19 Infection: A Case Report and a Literature Review

**DOI:** 10.3390/jcm12237407

**Published:** 2023-11-29

**Authors:** Wissam Al Rida Ayoub, Dina Lizzeik, Jana Berro, Sami Faddoul, Mohamad El Dassouki, Abdul Rahman Shatila, Moussa A. Chalah, Samar S. Ayache

**Affiliations:** 1 Lebanese American University Medical Center—Rizk Hospital, Beirut 1100, Lebanon; wissamalrida.ayoub@lau.edu (W.A.R.A.); jana.berro@lau.edu (J.B.); mohamad.dassouki@laumcrh.com (M.E.D.); abdulrahman.shatila@laumcrh.com (A.R.S.); 2 Doctors’ Center Radiology and Laboratory, Beirut 1000, Lebanon; sami.faddoul1@gmail.com; 3 Department of Neurology, Gilbert and Rose-Marie Chagoury School of Medicine, Byblos 4504, Lebanon; samarayache@gmail.com; 4 Institut de la Colonne Vertébrale et des NeuroSciences (ICVNS), Centre Médico-Chirurgical Bizet, 75116 Paris, France; 5 EA4391 Excitabilité Nerveuse & Thérapeutique, Université Paris Est Créteil, 94010 Créteil, France; 6 Department of Clinical Neurophysiology, DMU FIxIT, Henri Mondor University Hospital, Assistance Publique-Hôpitaux de Paris (APHP), 94010 Créteil, France

**Keywords:** COVID-19, SARS-CoV-2, Ramsay Hunt Syndrome, RHS, herpes zoster, Varicella Zoster Virus, herpes zoster oticus

## Abstract

(1) Background: COVID-19 infection has affected almost 6 million people worldwide. Geniculate Ganglion Zoster resulting in Ramsay Hunt Syndrome (RHS) has been rarely described in this context. (2) Methods: Here, a case of RHS in the context of asymptomatic COVID-19 infection is reported followed by a literature review of the previously published cases (PubMed research combining “COVID-19” and “Ramsay Hunt Syndrome” or their abbreviations/synonyms, searching for data published at any time till October 2023). (3) Results: Five cases have been previously published (age range: 25–67 years; *n* = 3 males). Three patients were known to be immunocompetent prior to infection, one was receiving corticotherapy for lung disease, and one had an unspecified immune status. RHS predominantly involved both facial and vestibulocochlear nerves, with one case exclusively involving the facial nerve as the presented case. Regarding facial nerve palsy, three were right-sided (like the current report) and two were left-sided. Two cases were asymptomatic to COVID-19 (like the present patient), one had mild fatigue, and two had classical COVID-19 symptoms preceding RHS symptoms. Workup included serological testing against Varicella Zoster Virus and PCR assays that can detect the viral DNA in saliva, blood, tears, exudates, and cerebrospinal fluid. The treatment combined antiviral and corticosteroid therapies which yielded heterogeneous outcomes that might be related to some demographic and clinical data. (4) Conclusions: RHS rarely occurs in the context of COVID-19. Early recognition is important. Management seems to be similar to the classical condition. Some data may help predict facial nerve recovery.

## 1. Introduction

COVID-19 disease caused by the severe acute respiratory syndrome coronavirus 2 virus (SARS-CoV-2) could result in a wide range of symptoms [1]. This includes mild respiratory symptoms, anosmia, hypogeusia, myalgia, other systems involvements, and even severe multi-organ failure with a high mortality rate [1]. Several neurological complications have also been reported with COVID-19 including neuropathies [1,2,3]. On one side, the latter symptoms could occur as a direct consequence of COVID-19 through some hypothetical mechanisms (e.g., autoimmunity, neurovirulence, neurotropism, neuroinvasion, ischemia of vasa nervorum) [2,3,4]. On the other side, it could occur secondary to the reactivation of latent viruses. In fact, the immune response triggered by COVID-19 infection could be at the origin of herpes virus reactivation (e.g., Varicella Zoster Virus (VZV)) in some cases [5,6]. It is also worth noting that herpes virus reactivation has been also described in the context of COVID-19 vaccination based on observational studies, case reports, and series, which makes it difficult to draw formal conclusions [7,8].

After primary exposure to chicken pox, VZV can remain dormant in spinal and cranial nerve ganglia, but it can be reactivated later on in specific contexts. 

Ramsay Hunt Syndrome (RHS) is a rare syndrome described by James Ramsay Hunt in 1907 [1,9]. It occurs in 0.3–18% of patients and is characterized by a reactivation of VZV—an alpha herpes virus causing chickenpox and herpes zoster (shingles)—in the geniculate ganglion. This results in facial palsy along with an erythematous vesicular or maculopapular rash in the distribution of the facial nerve sensory supply [10]. RHS sometimes involves the vestibulocochlear nerve (e.g., hearing loss, otalgia, tinnitus, vertigo) [1] or even the vagus nerve [10]. Some reports also evoke a plethora of other symptoms such as dysgeusia, dry eyes or mouth, dysarthria, hyperacusis, and tearing [10].

To the best of our knowledge, only a few reports have described RHS in association with COVID-19 infection [1,11,12,13,14] or 2–3 days after administration of the COVID-19 vaccine [15,16,17]. Setting the right diagnosis allows the delivery of proper management and optimization of the clinical recovery. 

Here, we report a patient who presented with RHS in the context of an asymptomatic COVID-19 infection. The case will be discussed in light of the limited available literature on the matter. 

## 2. Case Presentation

A 60-year-old man with a history of arterial hypertension and chickenpox infection during childhood was admitted to the emergency department with oral paresthesia, altered taste sensation (dysgeusia), right facial weakness, and right buccal and ear vesicular rash that started five days prior to presentation. The patient first developed an altered taste sensation which he described as an acidic taste. Three days later, a dark red rash appeared on the buccal mucosa. He then presented to the emergency department with upper and lower right facial weakness (difficulty with eye closure and difficulty smiling) suggesting peripheral facial nerve palsy. He had multiple crusted lesions on the right side of the face, on the periauricular and perioral area in the V3 distribution, and also on the right upper anterior neck. He had no other neurological deficits. He had no complaints of tinnitus, hearing impairment, or vertigo, and the neurological exam did not reveal any sign of vestibulocochlear involvement. The otoscopic examination revealed intact tympanic membranes and external auditory canals. 

The patient had no symptoms suggestive of COVID-19 infection (absence of fever, sore throat, cough, anosmia, fatigue, or other respiratory tract symptoms or signs on clinical exam).

No other signs of infectious, autoimmune, or systemic diseases were observed on the physical exam. 

The nasopharyngeal COVID-19 polymerase chain reaction (PCR) test was performed as part of the routine evaluation and came out positive. The patient received his third dose of the COVID-19 vaccine four months prior to presentation. A blood workup was performed showing high IgM and IgG varicella titers, 7.2 pg/mL (normal (NL) < 0.8 pg/mL) and >5000 IU/L (NL < 80 IU/L), respectively. PCR assays to detect VZV DNA (e.g., from the skin lesions) were not available. Herpes Simplex IgM titers were negative and IgG titers were positive. The CD4/CD8 ratio was 0.66 (NL 1.4–1.8). Complete blood count and differential (CBCD), erythrocyte sedimentation rate (ESR), C-reactive protein (CRP), electrolytes, liver enzymes, creatinine, urea, and glycemia were all within normal range. 

**Figure 1:**
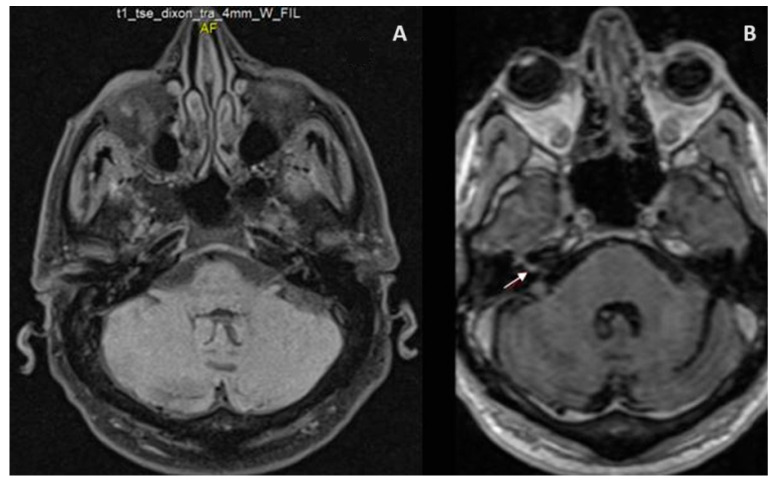
(**A**) Axial T1 of the brainstem at the level of exit of the facial nerve. (**B**) T1 post gadolinium image demonstrating 4 mm focal nodular enhancement in the fundus of the right internal auditory canal (white arrow).

A brain magnetic resonance imaging (MRI) scan showed a 4 mm focal nodular enhancement in the fundus of the right internal auditory canal (Figure 1).

These findings confirmed the diagnosis of geniculate herpes zoster or RHS in the context of asymptomatic COVID-19 infection. 

The patient was started on a 10-day course of valacyclovir 1000 mg PO three times daily along with prednisone 1 mg/kg/day PO for five days then tapered over 10 days. No antiviral therapy was considered to treat COVID-19 infection as the patient was asymptomatic for COVID-19. One month later, the patient had minimal improvement of right facial weakness with a total resolution of the rash.

The patient provided his written informed consent to publish his clinical data.

## 3. Discussion 

### 3.1. Available Case Reports of Ramsay Hunt Syndrome in the Context of COVID-19 

RHS affects 5/100,000 patients annually [18]. It typically manifests in people between the ages of 60 and 80 but can also affect people of any age between 19 and 89 years [18]. RHS encompasses a triad of peripheral facial palsy, vesicular rash over the pinna, and auricular pain. RHS could also manifest as a herpetiform rash and otalgia, associated with ipsilateral peripheral facial nerve palsy (i.e., like the current report), and, in the most severe forms, ipsilateral vestibulocochlear nerve involvement [9]. 

PubMed research looking for articles published at any time till October 2023 in the English language and combining the words “COVID-19” OR “SARS-CoV-2” AND “Ramsay Hunt Syndrome” OR “herpes zoster oticus” has identified five cases of RHS in the context of COVID-19 infection [1,11,12,13,14] and three cases of RHS following COVID-19 vaccination [15,16,17]. A summary of the case reports is available in Table 1 and Table 2.

### 3.2. Clinical Presentation of Ramsay Hunt Syndrome in the Context of COVID-19 Infection

Cases involving COVID-19 infections had an age range of 25–67 years and concerned both males (*n* = 3) and females (*n* = 2) [1,11,12,13,14]. Most of the previous publications involved patients who were known to be immunocompetent prior to their presentation [1,11,12], whereas one was receiving corticotherapy for interstitial lung disease [13], and one with unspecified status [14]. RHS predominantly involved both facial and vestibulocochlear nerves [1,11,12,13] with one case exclusively involving the facial nerve [14] as in our patient. Regarding facial nerve palsy, three were right-sided [12,13,14] (like the current report), and two were left-sided [1,11].

Similarly to our patient, one published case also presented taste impairment [11], a finding that can occur in the context of RHS [10]. In fact, one physiological study has shown that, compared to healthy controls, patients with RHS had reduced taste perception (based on electrogustometric thresholds) in the territories of the chorda tympani and the major petrosal nerve (branches of the facial nerve) as well as the glossopharyngeal nerve, altered taste function (examined using chemogustometry), and an anterior fungiform papillary atrophy (assessed via contact endoscopy) [19]. However, it is also noteworthy that taste impairment could occur in the context of COVID-19 infection [20]. In this context, involved hypothetical underlying mechanisms include damage or dysfunction of taste buds, salivary gland, olfactory epithelial cells, or cranial nerves, directly or via pathways involving Angiotensin-converting enzyme 2 receptors, Toll-like receptors, hypoxia, inflammatory response comprising cytokines as well as cellular and genetic changes, to cite a few [20]. However, it is unlikely that dysgeusia be explained by COVID-19 infection in our patient who is otherwise COVID-19 asymptomatic. 

Furthermore, among the other associated symptoms in published RHS and COVID-19 cases, one patient additionally presented with aseptic meningitis and Bruns nystagmus [12], another patient exhibited abducens nerve palsy contralateral to facial nerve palsy [13], and in one case report, concomitant ataxia at the time of presentation was described [14]. 

Chronologically, in the context of SARS-CoV-2 infection, herpes zoster rash onset varies across the available reports ranging between 2 days before and 70 days after the onset of COVID-19 symptoms, or in a simultaneous manner in very few patients [5]. In rare cases, the rash could be an indicator, or the first manifestation of COVID-19 infection [21,22]. Particularly, regarding COVID-19 symptomatology in published RHS cases, two were asymptomatic as the present case [13,14], one case only had mild fatigue during RHS recovery [11], and two had classical COVID-19 symptoms (i.e., sore throat and fever) preceding RHS symptoms by days [1] or weeks [12].

### 3.3. Pathophysiology of Ramsay Hunt Syndrome in the Context of COVID-19 

The pathophysiology underlying RHS is based on the reactivation of VZV which resides chronically in the geniculate ganglion. From a mechanistic perspective, the immune alteration in COVID-19 patients could be the trigger of herpes virus reactivation. A few cases of such a reactivation have been described in the context of COVID-19 infection and might be associated with older age, psychological stress, mechanical trauma, immunosuppression, lymphopenia, and cell-mediated dysfunction, to cite a few [3,23]. COVID-19 infection might constitute the stress factor for VZV reactivation [5]. Some authors also reported common comorbidities in patients who had herpes virus reactivation and COVID-19, namely hypertension followed by diabetes mellitus [5]. While herpes virus reactivation post-COVID-19 vaccine might be related to inflammation, autoimmunity, vaccine-induced hyperviscosity, vaccine-mediated hampering of the innate immunity targeting VZV, and the unavailability of VZV-specific CD8 cells due to the shift in naive CD8+ cells to target the vaccine [8,24,25,26], the underlying mechanisms action in the context of COVID-19 infection could involve various COVID-19-related T cell immune dysfunctions [27]. Here, it is worth noting that our patient had a low or inverted CD4/CD8 ratio which could reflect a suppression of the CD4+ T cells and/or an activation of the cytotoxic CD8+ T cells. Such a finding has been previously documented in the context of COVID-19 infection, but also in association with other conditions such as aging, chronic inflammation, metabolic or cardiovascular diseases, immune dysfunction, or immune senescence [28]. With regard to the latter condition, an association has been reported between COVID-19 diagnosis in patients above 50 years of age and increased risk of herpes zoster manifestation [29]. 

Among the mentioned variables, our patient relatively had an older age, he had a controlled hypertension, and exhibited low CD4/CD8 ratio. The possibility of immunosuppression in our patient and the previous reports cannot be formally ruled out in the absence of comprehensive evaluation. The reactivation of VZV could serve as an indicator of the individual’s declining immune response. In this context, additional investigations become necessary to assess the immune status. The causes of immunosuppression can be diverse and complex [30]. In the evaluation process, only a few possibilities have been ruled out based on medical history, clinical evaluation, and initial workup. A comprehensive assessment might have allowed formal conclusions to be drawn on the immune status, as well as providing further understanding of the pathophysiological mechanisms behind this manifestation. For instance, depending on the clinical context, some tests could help evaluating pathologies linked to immunosuppression such as infections (e.g., throat/skin swabs, CSF analysis, serum immunoglobulins, chest radiography/computed tomography, panculture), malignancies (e.g., blood smears, biopsies, imaging, myeloma workup), renal diseases (e.g., urinary albumin-to-creatinine ratio), pulmonary diseases (e.g., pulmonary function tests and imaging), antibodies and immune complements deficiency (e.g., antibodies and hemolytic assays), autoimmune diseases (e.g., antibodies and endocrine tests associated with connective tissue diseases), and genetic diseases (e.g., tests exploring diseases such as alpha-1 antitrypsin deficiency), to cite a few [30].

### 3.4. Evaluation of Ramsay Hunt Syndrome in the Context of COVID-19

Concerning COVID-19 diagnosis, all the published cases had an initial positive SARS-CoV-2 nasopharyngeal swab testing (rapid antigen test [1], PCR [11,12,13,14]), which sometimes turned negative upon retesting [1,12,14]. The routine laboratory workup was normal in some cases like our patient [1,11,12,13], including CBCD, ESR, CRP, renal and hepatic functions, blood glucose, and electrolytes. However, some cases had elevated white and red blood cell counts [13], marginally high CRP levels [1], and slightly elevated ferritin levels [13].

With regard to RHS diagnosis, PCR assays could detect HZV DNA in skin lesions, as well as saliva, blood mononuclear cells, tears, and cerebrospinal fluid (CSF) [31,32]. Only two of the five published cases assessed VZV PCR (i.e., CSF samples) and identified the viral DNA [12,14]. When PCR testing is not possible, a serological workup could be performed [32]. Blood serological testing of IgM and IgG against VZV can confirm the diagnosis such as in the present case and a previous case [12] (VZV IgG positive, VZV IgM not mentioned). The remaining three published cases did neither perform serological testing nor PCR assessment. The serological profile in the presented patient is compatible with a transient rise in IgM and an important memory IgG response. Along with the prior history of chickenpox infection, this suggests a VZV reactivation. 

CSF analysis was performed in some of the published cases and has yielded pleocytosis [12,13,14], high protein level [12,13,14], positive VZV PCR [12,14], and negative COVID-19 PCR [12]. Of interest, one study on herpes zoster has assessed the relationship between CSF VZV viral load, CSF biomarkers of nerve damage and astrogliosis, and patients’ outcomes [33]. Here, a significant correlation was found between viral load and biomarkers (i.e., neurofilament and glial fibrillary acidic proteins), suggesting an association between viral DNA and the amount of tissue damage. However, no correlations were found between the biomarkers and patients’ outcomes [33]. 

Brain neuroimaging is of limited diagnostic value [31]. Nevertheless, gadolinium-enhanced T1-weighted MRI might show an enhancement of the geniculate ganglion (as in our patient), the facial nerve, or the vestibulocochlear complex [34,35]. Brain MRI would also help in excluding tumors or demyelinating lesions [34,35]. Among the available publications on RHS and COVID-19, cerebral MRI was normal in one case [1], or showed facial nerve enhancement/inflammation [13,14] or mastoid effusion [12]. 

### 3.5. Management of Ramsay Hunt Syndrome in the Context of COVID-19 

The mainstay treatment of RHS is a combination therapy of steroids and antiviral therapy [36]. The treatment regimen used is oral prednisone (1 mg/kg/day over five days, tapered over 10 days) combined with 7–10 days course of either intravenous acyclovir (250 mg three times per day) or oral acyclovir 800 mg five times per day [34], while some authors used 5–7 valacyclovir 3000 mg/day [10]. The reported RHS/COVID-19 cases were treated with acyclovir (IV [12,14] or oral [1,11,13]) combined with corticotherapy in four of them [1,11,12,13]. Other treatments included vasodilators (xanthinol nicotinate) [1], vitamins (B1, B6, B12) [1], antibiotics [1], intratympanic corticosteroid (triamcinolone acetonide) [1], or eye care [11,13].

### 3.6. Clinical Outcomes and Prognostic Factors of Ramsay Hunt Syndrome in the Context of COVID-19

The clinical outcome in the presented patient was a minimal improvement of right facial weakness at one month. The outcome of RHS has been inconsistent. The available literature included a case of a complete facial nerve recovery on the fifth day of admission but with persistent hearing deficit for up to one month (with no available data on longer follow-up) [1], a case that improved at one month and fully recovered at two months [11], and two cases with persistent facial weakness like our patient (at least after one week of treatment [14] or at six months [13]) despite abducens and vestibular nerve recovery [13] or improvement in ataxia [14]. In one of the latter cases, facial asymmetry was accompanied by pronounced hearing loss at 6 months [13]. Interestingly, MRI facial nerve enhancement was found to last 1–6 months [12,13]. 

In terms of prognosis, facial nerve recovery could depend on several factors. Regarding sociodemographic data, while some data suggest no relationship between age, sex, and clinical outcome, better recovery seems to occur in young patients compared to those above 60 years of age according to other data [36,37,38,39]. With regard to baseline clinical data, the presence of metabolic diseases appears to contribute to the extent of facial nerve recovery [36]. In what relates to RHS per se, facial nerve recovery seems to be worse in the case of other cranial nerves involvement compared to isolated facial nerve palsy [34,37]. Other clinical prognostic factors seem to include oropharyngeal involvement (such as in our patient), high salivary viral load, dry eye, or lagophthalmos [40,41], the latter ophthalmic findings indicating an involvement of the large superficial petrosal nerve. 

Finally, as for the management, the time of treatment initiation appears to be important. In one study, 75% of patients had excellent prognoses if treatment was started within 72 h of symptom onset and 30% if treatment was initiated after seven days [34]. Nerve excitability testing showed lower nerve degeneration with early versus late management [34]. This highlights the importance of early diagnosis and management. A combination of antiviral treatment and steroids appears to be superior to steroid monotherapy in terms of facial nerve recovery [35], while the antiviral route of administration (e.g., acyclovir) seems to have no impact on the clinical outcomes [34]. The types of corticosteroids that are combined with acyclovir have been associated with different rates of recovery, with the highest complete recovery rates observed with methylprednisolone [36]. This could be explained by the higher anti-inflammatory effects and the higher affinity of the latter to glucocorticoid receptors compared to the other molecules [42].

### 3.7. Limitations

It is noteworthy that the present work has some limitations. First, concerning the reported case, a comprehensive workup would have allowed the determination of the patient’s immune status. Second, regarding the literature review, limitations could include the small number of reported cases worldwide, which limits the generalizability of the clinical presentation and outcomes to other similar cases, and potentially the selection and language biases, since only PubMed-indexed articles published in the English language were considered. However, this work highlights the rarity of RHS in the context of COVID-19 and the pertinence of early recognition and management of this clinical entity especially in atypical settings (i.e., absence of COVID-19 symptoms). 

## 4. Conclusions

RHS rarely occurs in the context of COVID-19 infection. Management seems to be similar to the classical condition combining antiviral and corticosteroid therapies. Some demographic and clinical data may help predict the outcomes in terms of facial nerve recovery. Early recognition and treatment appear to be crucial to optimizing clinical outcomes. 

## Figures and Tables

**Table 1:** A summary of the available case reports on Ramsay Hunt Syndrome in the context of COVID-19 infection.

Authors	López-Blanco et al., 2020 [14]	Mehta et al., 2021 [1]	Alonzo-Correa et al., 2021 [11]	Antonescu et al., 2021 [13]	Chu et al., 2023 [12]
**Demographic and clinical data**	67-year-old male (information NP)	32-year-old previously healthy male	25-year-old healthy female	54-year-old female receiving steroids	25-year-old immunocompetent male
**RHS clinical presentation**	Right facial paralysis, otalgia, headache, fever, right ear and soft palate skin rash, and gait ataxia	Left facial weakness, left pinna vesicular eruption, hearing loss, tinnitus, and difficulty in communication following COVID-19 symptoms; afebrile at presentation 20 days following symptoms onset	Left earlobe vesicular eruption and jawline/neck pain, followed by left hyperacusis (two days later) then left facial palsy/paresthesia, ageusia, and eye symptoms (one day later)	Acute vestibular syndrome followed by left abducens nerve palsy, right peripheral facial palsy, right ear vesicular rash (a few days later), and severe right hearing loss (one month later)	RHS and aseptic meningitis three weeks following COVID-19 symptoms: right facial palsy/hypoesthesia, right facial and mastoid tenderness, hearing impairment, headache, dizziness, vomiting, nystagmus, afebrile; followed by right ear vesicles
**COVID-19 presentation**	No clinical, laboratory, or neuroimaging evidence	Fever, sore throat	Fatigue; no fever, cough, dyspnea, or anosmia	No fever, cough, dyspnea, myalgia, or arthralgia	Fever, sore throat
**Investigations**	- CSF analysis: pleocytosis, high protein levels, positive VZV PCR - Brain MRI: inflammation of the right facial nerve - Nasal swab SARS-CoV-2 PCR initially positive then negative	- Normal routine blood tests, marginally elevated CRP, negative HSV serology - Brain MRI: normal - SARS-CoV-2 antigen test positive then PCR negative 10 days later	- Normal CBCD, Chem-7 test, CRP, ESR; negative HIV serology - SARS-CoV-2 PCR positive	- CBCD showing leukocytosis and high red blood cells and hemoglobin; normal CRP, renal, and hepatic tests, slightly increased ferritin; negative HIV, hepatitis B/C, and syphilis serology - Brain MRI: Enhancement in the right facial and vestibulocochlear nerves and slight enhancement of the left facial nerve - CSF: positive Pandy’s reaction, increased protein, and slight pleocytosis - SARS-CoV-2 PCR positive	- CSF analysis: elevated protein, pleocytosis, VZV positivity, and negativity for SARS-CoV-2 virus, Mycobacterium tuberculosis, and other pathogens (Meningitis/Encephalitis Panel)
**Management**	- Acyclovir IV for seven days	- Acyclovir 800 mg PO five times per day for 10 days - Dexamethasone 8 mg IV twice per day, Xanthinol nicotinate 1 g IV twice per day, Neurobion Forte IV once daily, antibiotic, triamcinolone acetonide 40 mg/mL intratympanic bilaterally 0.5 mL on day 1, 3, and 5 - Oral steroids afterward	- Acyclovir 4 g PO per day for 14 days - Dexamethasone 8 mg/2 mL IM then Prednisone 1 mg/kg/day PO with weekly tapering - Eye care (unpreserved 1.5% hyaluronic acid drops, 5% dexpanthenol gel, nightly hydroxypropyl methylcellulose ointment, and left eye patching)	- Acyclovir 4 g/day PO for seven days - Artificial tears	- Acyclovir 10 mg/kg IV every 8 h for 10 days - Prednisolone 1 mg/kg
**Outcomes**	Improvement in ataxia but persistence of facial paralysis	Mild improvement in tinnitus, complete resolution of facial weakness, speech discrimination score of 70% with bilateral hearing aid; no further changes at one month	Significant improvement following one month of treatment, recovery at two months	Significant improvement at six months (complete remission of vestibular and oculomotor symptoms, improvement but persistence of facial asymmetry, severe right hearing loss)	Alleviation of headache, vomiting, and dizziness; persistence of abnormal MRI signals at one month (right cochlea, semicircular canals, and facial nerve) suggesting unresolved facial palsy

CBCD: complete blood count with differential; CRP: C-reactive protein; CSF: cerebrospinal fluid; ESR: erythrocytes sedimentation rate; HIV: human immunodeficiency virus; HSV: herpes simplex virus; IgG: immunoglobulin; IM: intramuscular; IV: intravenous; NP: not provided; PCR: polymerase chain reaction; PO: per os; RHS: Ramsay Hunt Syndrome; VZV: Varicella Zoster Virus.

**Table 2:** A summary of the available case reports on Ramsay Hunt Syndrome in the context of COVID-19 vaccination.

Authors	Rodríguez-Martín et al., 2022 [15]	Woo et al., 2022 [16]	Lakhoua et al., 2022 [17]
**Demographic and clinical data**	78-year-old female with a history of childhood poliomyelitis and untreated arterial hypertension	37-year-old previously healthy male	65-year-old male with treated arterial hypertension; no history of chickenpox infection or varicella vaccine
**RHS clinical presentation**	Malaise, gait instability, nausea, right otalgia and vesicles/crusted lesions, right-side predominant hearing loss, right facial nerve palsy, and left nystagmus three days following COVID-19 vaccine (BNT162b2 mRNA)	Fever, right otalgia, right ear and canal vesicles, right hearing loss, vertigo, tinnitus, hearing loss, facial palsy, tongue numbness, and dysgeusia two days following the COVID-19 vaccine (BNT162b2 mRNA)	Vesicular eruption three days after the first shot of the COVID-19 vaccine (BNT162b2 mRNA), left hemifacial paralysis and pain, and cutaneous exacerbation one week after the second shot
**Investigations**	- Workup included blood tests, COVID-19 PCR test, brain CT scan, video head impulse test showing right vestibular hypofunction, and audiometry confirming bilateral right-side predominant hearing loss (left side presbycusis)	- Exudates PCR positive for VZV - Saliva negative for SARS-COV-2 - Brain CT scan: normal	Normal CBCD four days following the first shot; VZV antibodies not performed
**Management**	NP	NP	- Valacyclovir 100 mg PO twice per day for seven days - Pregabalin 75 mg PO 3 tablets per day for two months - Physiotherapy
**Outcomes**	Persistence of instability and hearing loss and slight improvement in facial paralysis at two weeks	NP	Improvement in facial paralysis within one-month, rash resolution at four months, persistence of pain/tingling at six months

CBCD: complete blood count with differential; NP: not provided; PCR: polymerase chain reaction; PO: per os; RHS: Ramsay Hunt Syndrome; VZV: Varicella Zoster Virus.

## Data Availability

All the data related to this publication are available within the article.
